# Spatio-Temporal Disparity and Driving Forces of the Supply Level of Healthcare Service in the Yangtze River Delta

**DOI:** 10.3389/fpubh.2022.863571

**Published:** 2022-04-21

**Authors:** Zaijun Li, Meijuan Hu

**Affiliations:** ^1^Research Institute of Central Jiangsu Development, Yangzhou University, Yangzhou, China; ^2^College of Tourism and Culinary Science, Yangzhou University, Yangzhou, China

**Keywords:** the supply level of healthcare service, spatio-temporal evolution, spatio-temporal association, geographically and temporally weighted regression (GTWR), Yangtze River Delta

## Abstract

The equalization of healthcare supply is not only related to the people's need for a better life, but can also provide a strong guarantee for the high-quality and sustainable development of the Yangtze River Delta integration. By using exploratory spatial analysis techniques, this study analyzed the spatio-temporal evolution characteristics and heterogeneous influence effects of the supply level of healthcare service in the Yangtze River Delta from 2007 to 2019. It was found that the supply level of healthcare service in the Yangtze River Delta had improved significantly. The differences in the supply level of healthcare service between cities had tended to narrow without polarization, and the supply level of healthcare service generally showed a high spatial pattern in the south delta and low spatial pattern in the north delta. The higher the supply level of healthcare service was, the weaker the interannual variability was. The supply level of healthcare service in the Yangtze River Delta region presented obvious spatial association and differentiated tendency of local high and low spatial clusters. The relative length and curvature of the supply level of healthcare service in the Yangtze River Delta generally presented a spatial pattern with low values in the northeast and high values in the southwest. Population density and urban-rural income gap generally exhibited negative spatio-temporal impact on the supply level of healthcare service across most cities. On the other hand, urbanization level and per capita disposable income generally had positive spatio-temporal impact on the supply level of healthcare service across most cities. Per capita gross domestic product (GDP) showed an increasingly positive spatio-temporal impact on the supply level of healthcare service across most cities. While per capita fiscal expenditure exhibited significantly negative impact on the supply level of healthcare service across most cities in space.

## Introduction

Healthcare service provision is an important part of basic public service and a major livelihood project related to people's health (1, 2). According to the 2018 Blue Book of Public service, healthcare has become the essential public service component that has attracted the most public attention in recent years. With the main contradiction facing the shift of China's society to the contradiction between unbalanced and insufficient development and the people's growing need for a better life, comprehensively improving the capacity and quality of basic public service provision is an inevitable requirement for safeguarding and improving people's wellbeing and promoting people's all-round development. In addition, it is an important guarantee for meeting people's ever-growing needs for a better life and building a moderately prosperous society. Despite rapid expansion of public service expenditures in China, there is growing disparity in access to healthcare service among residents in different regions due to differences in social and economic conditions (3, 4). The unbalanced development of basic healthcare service affects the flow of various factors within the region and limits the coordinated regional development. Therefore, it is necessary to clarify the spatial equilibrium degree and evolutionary trends of healthcare service, diagnose its potential influencing factors, and make recommendations to break through the development bottleneck and narrow the regional gap in the supply of healthcare service.

The Yangtze River Delta is one of the regions with the most active economic development, the highest degree of openness, and the strongest innovation ability in China. Over the years, with the implementation and promotion of the Yangtze River Economic Belt Development Plan and the Yangtze River Delta City Cluster Development Plan, the markets in the Yangtze River Delta region have been gradually integrated. In addition, the level of economic integration has been increasing. In 2019, the Yangtze River Delta Regional Integrated Development Outline was introduced, elevating the integrated development of Yangtze River Delta region to a national strategy, ushering new opportunities and challenges. Currently, the integrated development of the Yangtze River Delta is entering a period of accelerated advancement with high quality as the core. However, it cannot be ignored that the integrated development of the Yangtze River Delta is facing bottleneck constraints. Rapid economic development has accelerated intra-regional differentiation and increasingly uneven development among cities, especially in the area of basic public service, which is contrary to the goal of the Yangtze River Delta Regional Integrated Development Outline which alleges that by 2035, public service will be more universally shared, the standard system for basic public service will be basically established, the initial equalization access to basic public service will be achieved, the capacity and quality of non-basic public service supply will be comprehensively improved, and that people's basic livelihood security level will be roughly equal.

With the continuous promotion of the new medical reform, the overall supply level of healthcare service in the Yangtze River Delta city cluster has significantly improved, but there are still significant differences between regions, urban and rural areas, and populations. Identifying inequality trends and influencing factors is an urgent prerequisite in the process of promoting the balanced development of healthcare service supply and achieving higher quality integration in the Yangtze River Delta region. Therefore, this article aims to clarify the development status and regional differences in healthcare service supply among 41 cities in the Yangtze River Delta region, analyze the influencing factors of healthcare service supply in each city, and, on this basis, explore feasible paths to promote the coordinated development of healthcare service supply in the Yangtze River Delta region.

The remainder of this article is organized as follows. In Literature Review, the literature related to the subject of our research is introduced. Methodology and Data Sources introduces methods and data source, the multidimensional evaluation index of healthcare service supply level, the spatial analysis methods, and the data sources. Results presents the results. Finally, Discussion provides the discussion and conclusion of the study.

## Literature Review

Public good is one of the important attributes of healthcare resources. The supply of public goods needs to balance efficiency and equity, with a combination of planning and market approaches, and the same is true for the supply of healthcare resources. Extensive research are conducted on the equity of healthcare resource delivery in terms of the equity of healthcare financing, the accessibility of healthcare service, and the distribution of healthcare professionals (5–10). Arcaya et al. systematically reviewed the methodological, theoretical, and philosophical aspects of health inequalities, which provides scientific guidance for the study of health (11). To better assess the equity of healthcare service, a series of indicators, such as the Kakwani index (12, 13), the concentration index (CI) (14, 15), and the horizontal inequity (HI) (16, 17), are introduced to measure inequity in healthcare resource utilization and determine the contribution of different influences to the overall unfairness. Moreover, machine learning techniques are increasingly used to optimize health and social systems to ensure equality in healthcare delivery outcomes, performance, and resource allocation (18, 19). In addition, geographical distance is an important factor affecting the spatial accessibility of healthcare service, and geographical accessibility is recognized as an important component in assessing the overall accessibility of healthcare service and a fundamental goal in meeting residents' health needs. With the rise of the geographic information system (GIS), spatial analysis of healthcare demand, allocation, utilization, and accessibility in the healthcare delivery process has been extensively studied (20–23).

Efficiency and productivity are an important goal pursued by health policy makers and health systems due to scarce resources, vertical and horizontal competition, and health insurance and healthcare reforms (24). With the increased flexibility and generality of the nonparametric data envelopment analysis (DEA) and the Malmquist total factor productivity index, both static efficiency and dynamic productivity in the allocation and use of healthcare resources can be effectively measured, which helps guide management efforts to improve efficiency and reduce healthcare costs. Sherman firstly introduced the DEA model to identify and measure hospital efficiency (25). Thereafter, the evaluation of healthcare efficiency has been further advanced from multiple dimensions and perspectives. In terms of indicator selection, both micro- and macro-level data sets are used to assess the relative efficiency or productivity of the multi-input and multi-output healthcare sector (26–28). In terms of measurement methods, a super-slack-based measure (SBM) was introduced to assess the efficiency of the healthcare system in order to avoid the indistinguishable efficiency rank of decision-making units (DMUs) (29, 30). In addition, a two-stage network DEA was developed to evaluate whether there is a trade-off between service production and service quality in the healthcare delivery process (31, 32). In terms of research scales, related studies have focused on comparative analyses at the inter-country level (33, 34), inter- and intra-provincial level (35, 36), inter- and intra-city level (37), and township (38).

The development level of healthcare service is affected by a myriad of socioeconomic and environmental factors, such as economic development level, financial policy, and urbanization level (39–43). Based on data availability, six control variables were selected to detect the direction and degree of their impact on the supply level of healthcare service. Generally, rich cities tend to have developed healthcare service, advanced medical technology, and a reasonable amount of health resources, which increases the demand for medical and health goods and promotes the government's ability to supply healthcare resources (44). The ratio of total health expenditure to GDP as an important indicator of a city's health financing capacity reflects the relationship between health investment and socio-economic development. Particularly, the more developed the economy, the higher this ratio is (45). Per capita GDP (Pgdp) is selected to detect the impact of regional economic development level on the supply level of healthcare service.

Supply-side public goods supply theory suggests that a city's financial resources are an important determinant of the level of public goods supply (46). Sufficient financial resources provide solid foundation for local government to make larger investments in health resource, but with increased fiscal autonomy and competition for fiscal expenditure from other industries, the government is more willing to invest in higher-yielding industries, such as finance and real estate, which may reduce the intensity of investment in the health industry (40, 47). In this study, per capita fiscal expenditure (Pfin) is used to characterize the impact of fiscal autonomy.

Generally, the denser the population per unit area in a city, the greater the socio-economic effect of the scale of government public expenditure will be, thereby contributing to promoting the efficiency of government public service, improving the supply of primary healthcare service, and facilitating the organization and consumption of networked healthcare service (42, 48). Besides, the developed healthcare service in a given city not only meet the basic needs of local people, but also attract part of the population from other regions where local healthcare service cannot solve their issues (43). However, if population growth is faster than the supply level of healthcare resources, an increase in population density (Dens) may show a negative correlation with the supply of healthcare resource.

The urbanization process requires infrastructure and related public service for support. As the level of urbanization increases, the supply of health infrastructure as a public good for people's livelihood will increase, and the accessibility of health service will improve accordingly. Urbanization not only raises the level of primary healthcare service, but also facilitates the development of high-quality medical and health service (36, 49). Hence, urbanization contributes to promoting the supply of healthcare service. The ratio of urban population to permanent population represents the level of urbanization (Urba).

The demand and accessibility for local public service is expected to vary with income (48, 50, 51). In general, there is a pro-rich inequity in healthcare service delivery, which tends to favor wealthy residents and widens health status gap between urban and rural residents due to income and healthcare utilization gap (5, 52). Therefore, income is an important factor influencing the allocation of healthcare resources. Increased levels of economic development have contributed to increases in GDP per capita and population income, which in turn has raised the demand for high-quality healthcare service and promoted the efficient growth of medical and health resources. In this study, per capita disposable income (Disp) is attained by dividing total population with urban and rural residents' disposable income.

Due to socioeconomically disadvantages in rural areas and the urban bias in the supply of medical and health service, residents in rural and remote communities generally experience poorer health outcomes than many urban residents (53, 54). In addition, bias in urban fiscal, tax, investment, and social welfare policies can widen the urban-rural income gap by widening the health human capital gap between urban and rural residents (55, 56), which is not conducive to equitable access to primary health care service and exacerbates the maldistribution of healthcare resources between urban and rural areas. Narrowing urban-rural disparities is important to ensure equity in access to health service and in improving rural residents' health outcomes. In this study, urban-rural income gap (Inru) is represented with the income ratio of urban residents to rural residents.

As the first law of geography describes that everything is related to everything else, and near things are more related than distant things (57, 58), with the more and more obvious spatial trend of humanities and social sciences and the increasingly mature spatial econometric analysis methods, more and more scholars begin to pay attention to spatial dependence, spatial heterogeneity, spatial clustering, and other spatial effects in the study of regional economic growth (59), environmental and resource efficiency (60), innovation development (61), ecological welfare (62), etc. Moreover, spatial dependence is introduced to identify local spatial cluster and outlier effects of healthcare events (63), healthcare service industry (43, 64), and healthcare facilities (65, 66). Although the development of healthcare in the Yangtze River Delta cities is somewhat spatially dependent due to their geographical proximity and similar socioeconomic conditions, the supply of healthcare service is deeply influenced by multidimensional factors that vary considerably across regions and time periods. Hence, it is necessary to use dynamic local indicator of spatial association (LISA) statistics to reveal the spatio-temporal evolution process of healthcare service supply and to examine the driving effects of healthcare service supply by using the geographically temporally weighted regression (GTWR) model.

To sum up, related aspects in the definitions, concepts, and theories of health inequalities, measurement of the supply level of healthcare service, the supply efficiency of healthcare service, and the influencing factors of the supply level of healthcare service has been extensively studied. However, there are still some deficiencies. First, previous studies have typically used a single medical or health service indicator to describe the supply level of healthcare service, which may introduce biases in the spatially balanced measurement of the supply level of healthcare service. Therefore, the four combined indicators with the same weight are more appropriate to describe the supply level of healthcare service. Secondly, few research have explored the spatio-temporal evolution patterns and spatio-temporal association characteristics of the supply level of healthcare service and quantified the spatio-temporal heterogeneity influencing effects on the supply level of healthcare service in different cities. Thirdly, existing studies have focused on large-scale global and national comparative analyses. As far as the study of regional differences in healthcare service supply is concerned, there is not enough research on urban clusters or economic zones. These questions deserve close attention, which is not only important for the synergistic promotion of equalizing the supply of medical and health resources and constructing a demonstration region for high-quality development, but also helps to provide lessons for less developed urban clusters.

## Methodology and Data Sources

### Kernel Density Estimation

As a nonparametric estimation method without requiring assumptions about the form of the data distribution, the kernel density estimation (KDE) function can better describe the temporal distribution density of the sample data and discover continuity changes in convergence, polarization, and other related information (67). The KDE is expressed as follows:


(1) $$\begin{array}{l} f\left(x\right)=\frac{1}{nh}\overset{n}{\underset{i=1}{\sum }}k\left(\frac{x-x_{i}}{h}\right) \end{array}$$

where *f*(*x*) is the KDE value of healthcare supply level in *n* cities; *x*_*i*_ denotes the sample attribute being estimated; *n* is the number of spatial units; *h* is the bandwidth; and *k*denotes the weighted kernel function, including types of Gaussian kernel, Gaussian kernel, Triangular kernel, and Quartic kernel. In this study, the Gaussian kernel function is selected to fit temporal variation plot of healthcare service.

### Dynamic LISA

Dynamic LISA can be identified as the transfer of values in the static LISA scatterplot over a continuous-time trajectory (59). LISA is used to measure the degree of spatial differences between a region and its surrounding regions. It is calculated as follows:


(2) $$\begin{array}{l} I_{i}=Z_{i}\overset{n}{\underset{i=1}{\sum }}w_{ij}Z_{j} \end{array}$$

where *Z*_*i*_ and *Z*_*j*_ is the normalized values of unit *i* and *j* and, *w*_*ij*_ is the spatial weight. LISA divides the local spatial association into four types. The High-High (HH) and Low-Low (LL) types represent a positive local spatial autocorrelation, indicating a city and its surrounding cities with similar high or low values, while the High-Low (HL) and Low-High (LH) types indicate negative local spatial autocorrelation with diverging high or low adjacent values.

Dynamic LISA can be decomposed into relative length and curvature according to its geometric characteristics. The relative length of dynamic LISA (${\overset{\tilde{\;}}{N}}_{i}$) is calculated as (68, 69):


(3) $$\begin{array}{l} {\overset{\tilde{\;}}{N}}_{i}=\frac{N*\sum_{t=1}^{T-1}d\left(L_{i,t},L_{i,t+1}\right)}{\sum_{i=1}^{N}\sum_{t=1}^{T-1}d\left(L_{i,t},L_{i,t+1}\right)} \end{array}$$

where *N* is the number of spatial units, *T* is the annual time interval, *L*_*it*_ is the LISA scatterplot coordinate of the spatial unit *i* at time *t*, *d*(*L*_*i, t*_, *L*_*i, t*+1_) is the displacement distance of spatial unit *i* from time *t* to *t*+*1*. The greater the relative length, the more dynamic the local spatial dependence and local spatial structure of the supply level of healthcare service, and vice versa.

The curvature (*D*_*i*_) of dynamic LISA is calculated as follows:


(4) $$\begin{array}{l} D_{i}=\frac{\sum_{t=1}^{T-1}d\left(L_{i,t},L_{i,t+1}\right)}{d\left(L_{i,t},L_{i,t+1}\right)} \end{array}$$

If the relative displacement of LISA in city *i* is more curvaturous than the average, then *D*_*i*_ is >1. A larger *D*_*i*_ indicates that the supply level of healthcare service in a city has more volatile local spatial dependence direction. Conversely, a smaller *D*_*i*_ indicates the supply level of healthcare service in a city that has more stable local spatial structure.

### Geographically and Temporally Weighted Regression

The GTWR model can effectively deal with spatial and temporal nonstationarities simultaneously by introducing a temporal dimension on the basis of considering spatial heterogeneity (70), which helps to capture the spatially varying relationship between the supply level of healthcare service and the influencing variables in different years. It is calculated as (71):


(5) $$\begin{array}{l} Y_{i}=\beta_{i}\left(u_{i},v_{i},t_{i}\right)+\sum_{k}\beta_{k}\left(u_{i},v_{i},t_{i}\right)X_{ik}+\varepsilon_{i} \end{array}$$

where β_*i*_is the constant parameter of each space-time location *i* with the spatio-temporal coordinates of (*u*_*i*_, *v*_*i*_, *t*_*i*_), and β_*k*_ is the estimated coefficients of a series of independent variables *X*_*k*_ at location *i*.

### Data Sources

The supply level of healthcare service was calculated as follows: the number of healthcare institutions per capita, the number of healthcare beds per capita, the number of healthcare doctors per capita, and the healthcare expenditure per capita were first respectively calculated using the resident population; then, dimensionless processing was carried out before the equal weight was finally used to add the four indicators. The final equal weighted summed metric is used to characterize the supply level of healthcare service. All datasets used in determining the supply level of healthcare service and related independent variables were obtained from Zhejiang Statistical Yearbook (2008–2020), Jiangu Statistical Yearbook (2008–2020), Anhui Statistical Yearbook (2008–2020), China City Statistical Yearbook (2008–2020), and dedicated statistics website https://insights.ceicdata.com/. To avoid the inflation effect, economic data, such as GDP, income, and financial expenditure were converted based on the year 2007.

## Results

### Temporal Evolution of the Supply Level of Healthcare Service

**Figure 1:**
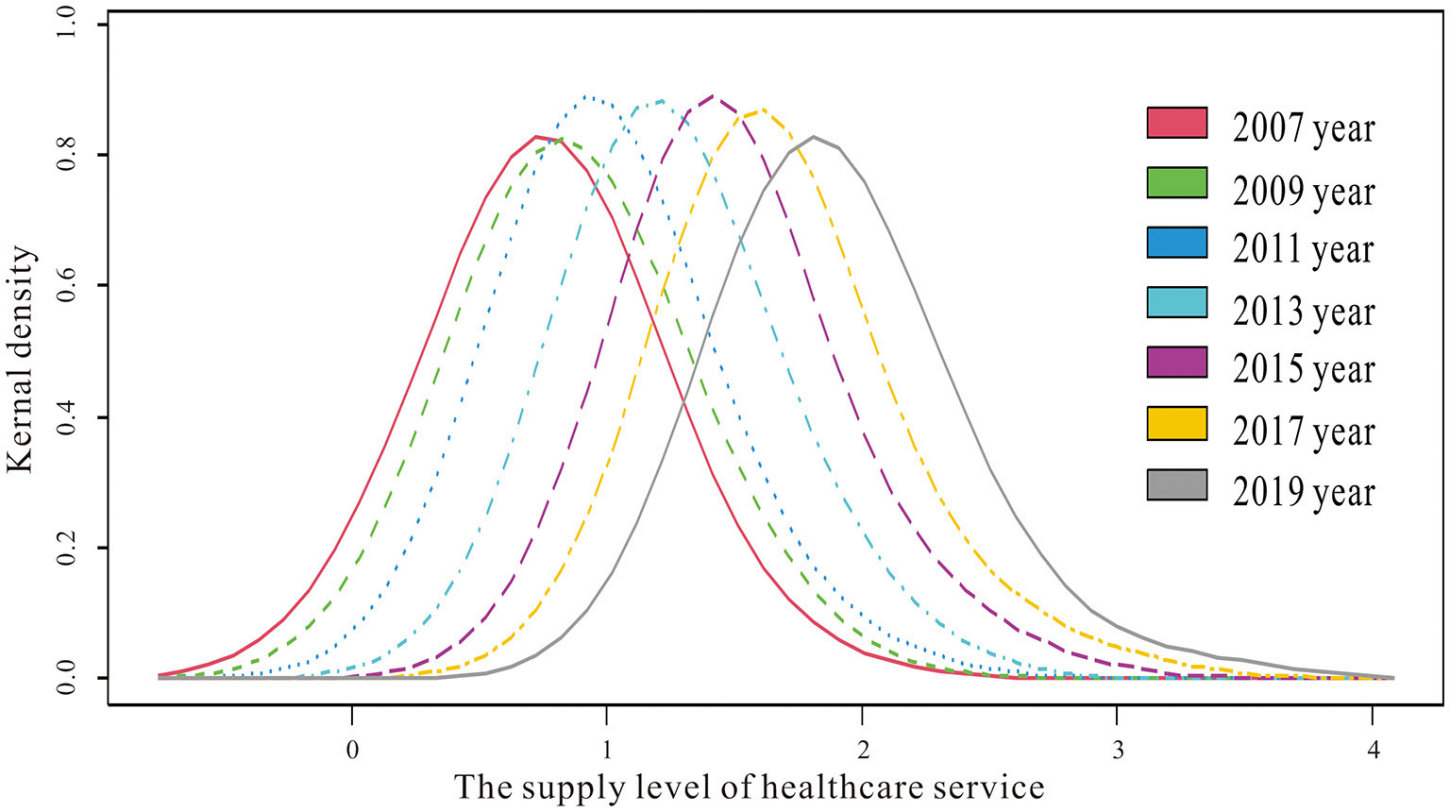
Kernel density estimation of the supply level of healthcare service.

The kernel density estimation method was firstly used to examine the spatial equilibrium state of the supply level of healthcare service in Yangtze River Delta region from 2007 to 2019, as shown in Figure 1. In terms of location, the peak value of kernel density exhibited an obvious displacement from 2007 to 2019. Over time, both the kernel density curve and the curve peak value of the supply level of healthcare service moved to the right, and the kernel density value changed from.8 in 2007 to 1.9 in 2019. In terms of shape, the kernel density curve maintains a single and a wide peak from 2007 to 2019. This indicated that the supply level of healthcare service in Yangtze River Delta was gradually improving and that the differences between cities tended to narrow without polarization.

**Figure 2:**
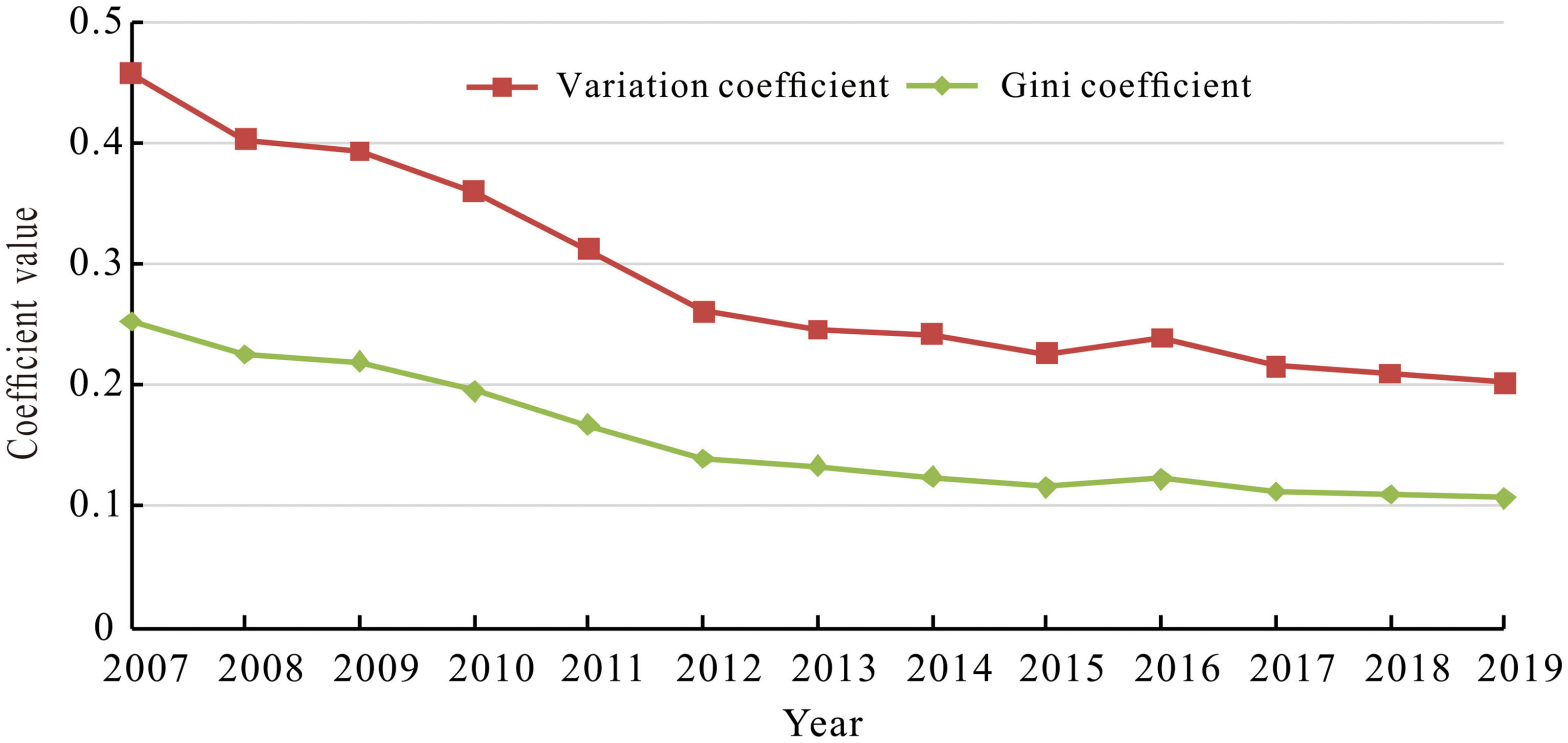
The variation and Gini coefficients of the supply level of healthcare service.

To further determine the variation of the supply level of healthcare service between cities, the CV and Gini coefficients of the supply level of healthcare service were calculated, as shown in Figure 2. The coefficient of variation (CV) and Gini coefficients of the supply level of healthcare service exhibited a similarly continuous downward trend. This was due to that with the rapid economic development in the Yangtze River Delta region, local governments paid more attention to optimizing the allocation of healthcare resources, improving the supply efficiency and level of basic healthcare service and achieving the balance between supply and demand of healthcare service. Moreover, with the advancement of the integration process of the Yangtze River Delta, inter-regional healthcare cooperation had gradually evolved from bottom-up fragmented cooperation to top-down systematic coordination. Currently, cooperation mechanisms had been established in medical treatment and control, medical and health information, joint prevention and control of infectious diseases, blood emergency support, trans-local large-scale activities, and other aspects.

**Figure 3:**
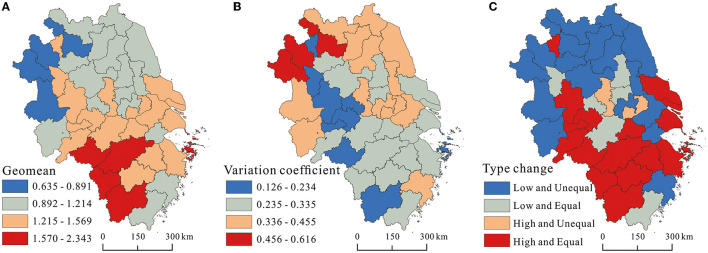
The coefficient of variation (CV) and Gini coefficient of the supply level of healthcare service.

Moreover, to differentiate the inter-annual variation of the supply level of healthcare service for each city, the annual geometric mean value and variation coefficient of healthcare service supply level from 2007 to 2019 was calculated. Specifically, taking 1.27 as the dividing line of high and low supply level of healthcare service and using.32 to separate relative equality and inequality groups, the temporal differentiation evolution types of the supply level of healthcare service for each city was visualized through ArcGIS 10.8 software, as shown in Figure 3A. The annual geometric mean of the supply level of healthcare service generally disclosed a distribution pattern of “high in the south and low in the north.” High level cities were highly concentrated around the south of Anhui province, the south of Jiangsu province, and the north of Zhejiang province, while low level cities were extensively scattered in the north of Anhui province and Jiangsu Province.

The variation coefficient for each city from 2007 to 2019 was calculated to identify their inter-annual fluctuation patterns. As shown in Figure 3B, there was a great difference in the variation coefficient among cities, with a fluctuation range from 0.126 to 0.616. Besides, the spatial pattern of the variation coefficient was nearly opposite to the geometric mean pattern of the supply level of healthcare service. The cities with relatively high variation coefficients were concentrated in the north of the delta and the south of Jiangsu province, especially Suzhou, Bozhou, and Fuyang in Anhui province, while the cities with relatively low variation coefficients were mainly distributed in the central and southern cities of Anhui province and all cities except Taizhou of Zhejiang province.

The combination differentiation pattern of high-low and equal-unequal levels of healthcare service provision was generally consistent with the geometric mean pattern of healthcare service provision (Figure 3C). The high and equal type mainly gathered in southern delta and scattered in Huaibei city, accounting for 39.02% of all types. The low and inequal type mainly surrounded around the northern delta and scattered in Taizhou, Suzhou, and Changzhou in Jiangsu province, accounting for 41.46% of all types. It can be found that the higher the supply level of healthcare service was, the weaker the interannual variability was and the more likely to form the cumulative inertia effect. In contrast, cities with lower supply level of healthcare service were more likely to go through large interannual fluctuations because they were greatly affected by the expenditure of healthcare service and reallocation of healthcare resources.

### Spatial Association Pattern of the Supply Level of Healthcare Service

To detect whether there was a spatial association between the supply level of healthcare service in the Yangtze River Delta, Moran's I was used to diagnose the spatial agglomeration phenomenon (Table 1). As listed in Table 1, The Moran's I of healthcare service supply levels from 2007 to 2019 were significantly positive, and the Z-score value was greater than the critical value of the normal distribution function at the 10% level. This indicated that the agglomeration phenomenon of healthcare service supply levels in the Yangtze River Delta was not randomly generated. The healthcare service supply levels in prefecture-level cities have obvious positive spatial association, and the observed values of neighboring regions have obvious clustering characteristics. In terms of development trend, Moran's I values showed a downward trend from 2007 to 2012, indicating that the supply level of healthcare service in the Yangtze River Delta tended to develop in a balanced manner. From 2012 to 2019, Moran's I values showed an increasing trend, indicating that the supply mobility of healthcare service in the Yangtze River Delta accelerated and the supply concentration of healthcare service increased constantly.

**Table 1:** The Moran's I statistics of the supply level of healthcare service.

**Year**	**2007**	**2008**	**2009**	**2010**	**2011**	**2012**	**2013**	**2014**	**2015**	**2016**	**2017**	**2018**	**2019**
Moran's I	0.3580	0.2828	0.2144	0.1662	0.1304	0.1049	0.1590	0.2272	0.2943	0.3206	0.3043	0.2888	0.2502
Z	3.8061	3.0314	2.3657	1.9178	1.7192	1.6965	1.8316	2.5515	3.2348	3.4977	3.3560	3.2131	2.8063
*p* value	0.0001	0.0012	0.0090	0.0276	0.0783	0.0974	0.0635	0.0054	0.0006	0.0002	0.0004	0.0007	0.0025

**Figure 4:**
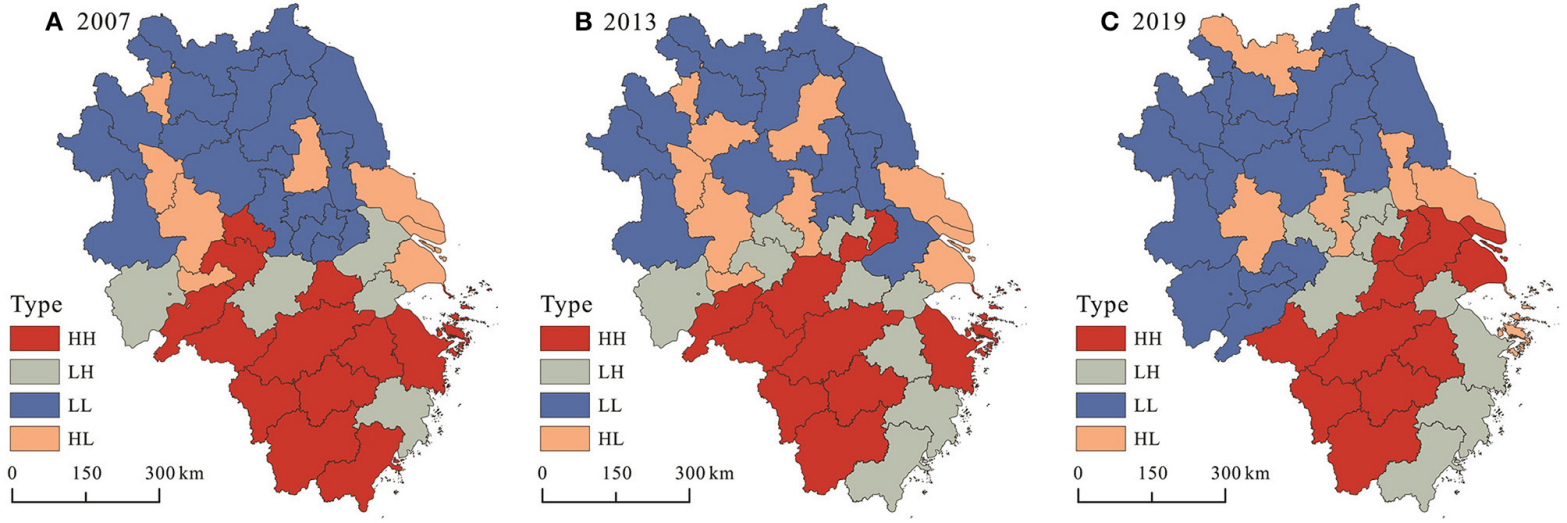
Local spatial association pattern of healthcare service supply in **(A)** 2007, **(B)** 2013, and **(C)** 2019.

To detect whether there was a cluster of observed values with similar or different characteristics in local areas, the local Moran's I index was used to reveal the local clustering characteristics of healthcare service supply levels. As shown in Figure 4, the supply level of healthcare service generally showed an obvious high and low spatial club differentiation. In terms of quantity, cities belonging to the LL type were the most in number, accounting for 39.02%, 31.71%, and 41.46% of the total number of cities in 2007, 2013, and 2019, respectively. Cities belonging to the HH type ranked second, accounting for 31.71, 24.39, and 24.39% of the total number of cities in 2007, 2013, and 2019, respectively. In contrast, the number of LH and HL type cities was relatively small. In terms of spatial distribution, it can be found that the HH type cities were mainly clustered in the south of the delta, the LL type cities were concentrated in the north of the delta, while the LH and HL type cities were distributed between or at the edge of HH and LL types. In terms of changes in spatial patterns, the LL type tended to increase and expand its spatial coverage, the HH type tended to move toward southern Jiangsu and other developed cities, the LH type tended to increase and contract toward coastal cities in Zhejiang province, and the HL type tended to decrease and remained stable in a few cities in the middle of the central delta.

**Figure 5:**
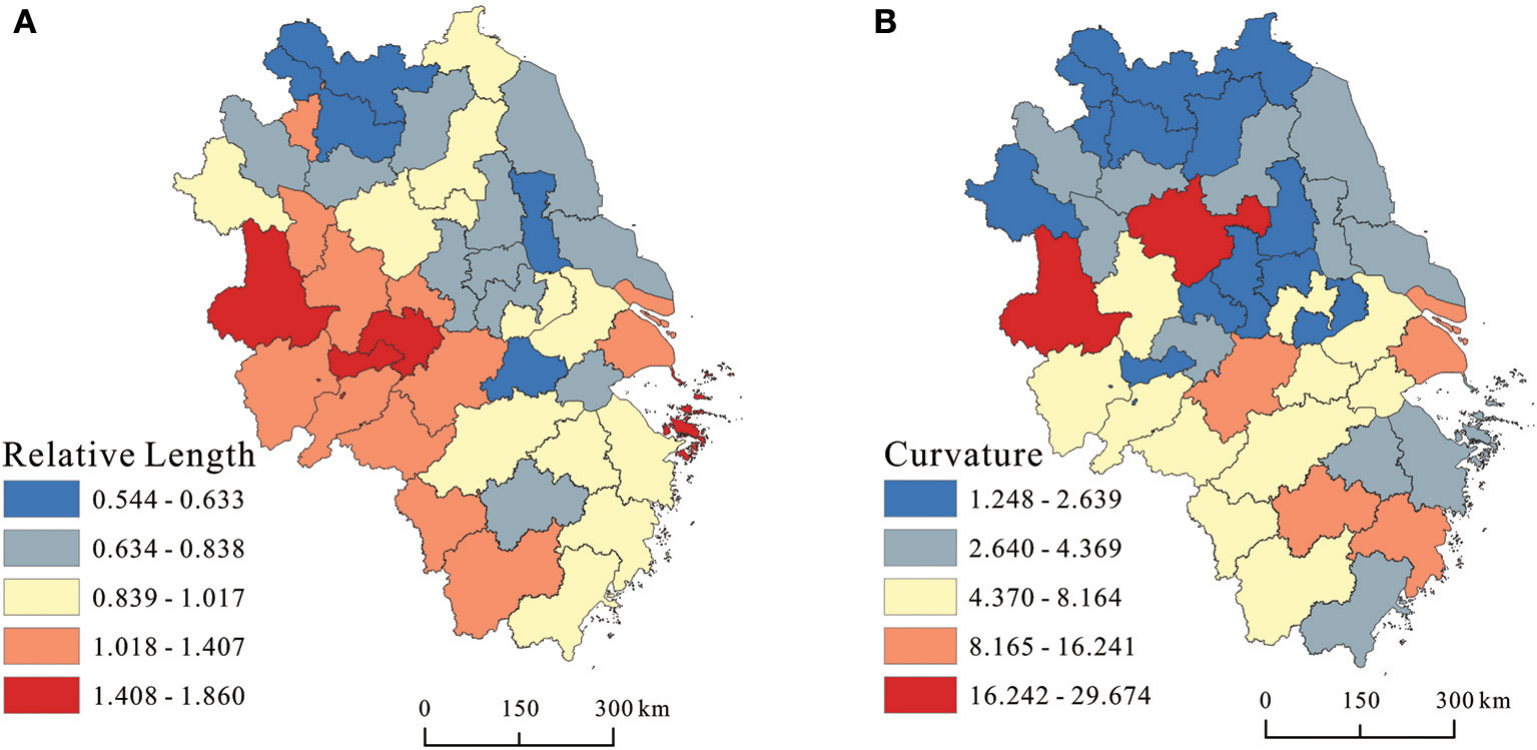
Spatial evolution characteristics of healthcare service supply levels in **(A)** relative length and **(B)** curvature.

The geometric characteristics of LISA time paths in each city was further calculated to reflect the dynamic changes of local spatial dependence. Using Natural Jenks for visualization, as shown in Figure 5, the cities with larger relative lengths were mainly concentrated in the southwest of Anhui province and scattered in Shanghai, Zhoushan, Lishui, and Quzhou. These cities were generally the hotspots of healthcare resources supply in Yangtze River Delta region, and the supply level of healthcare resources was developing rapidly. Cities with small relative lengths were concentrated in the northern Jiang province and coastal regions of Jiang province, indicating that the improvement of healthcare resources supply level in these cities was relatively lagging. The relative length of Nanjing city was relatively small due to the rapid growth of population that exceeded the supply of healthcare resources.

The curvature of most cities in northern Jiangsu and northern Anhui was relatively low, indicating that the development of healthcare resources supply had a relatively stable spatial dependence and spatial variability direction, that is, a strong spatial locking effect. The cities with higher curvature cities were mainly distributed in the middle of the delta, especially in Lu'an and Chuzhou city, which had the highest curvature, indicating that the supply of local healthcare service had a strong variability and lacked the incentive to continuously improve the level of healthcare resource supply, that is, government finance or external investment did not have continuity and lacked a strong regional radiation-driven growth pole.

### Local Varying Effects of Influencing Variables on the Supply Level Healthcare Service

**Figure 6:**
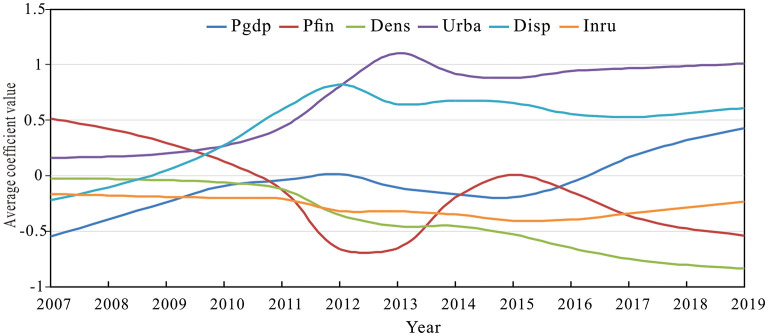
Temporal varying effects of different variables on the supply level of healthcare service.

The procedure developed by Huang et al. (70) was used to estimate the geographically and temporally weighted regression model using Matlab 18.0 software. The optimal bandwidth obtained by a function based on Gaussian distance decay was 0.3959 and the fitted adjusted R-squared was 0.9628. The annual average impact coefficients of each variable were calculated and plotted to reflect the temporal fluctuations of each variable. As shown in Figure 6, the impact coefficient of Pgdp was negative until 2017, except for 2012, and rose rapidly after 2017. Since 2011, the impact coefficient of Pfin gradually changed from positive to negative. The impact coefficient of Dens remained negative, and the intensity of the negative impact tended to increase. The impact coefficient of Urba remained positive and the intensity of the positive impact tended to increase. With the exception of 2007 and 2008, the impact coefficient of Disp remained positive, while the intensity of impact climbed rapidly from 2009 to 2012 and tended to fluctuate smoothly between 2013 and 2019. The impact coefficient of Inru remained negative, and the intensity of negative impact generally showed smooth fluctuations.

**Figure 7:**
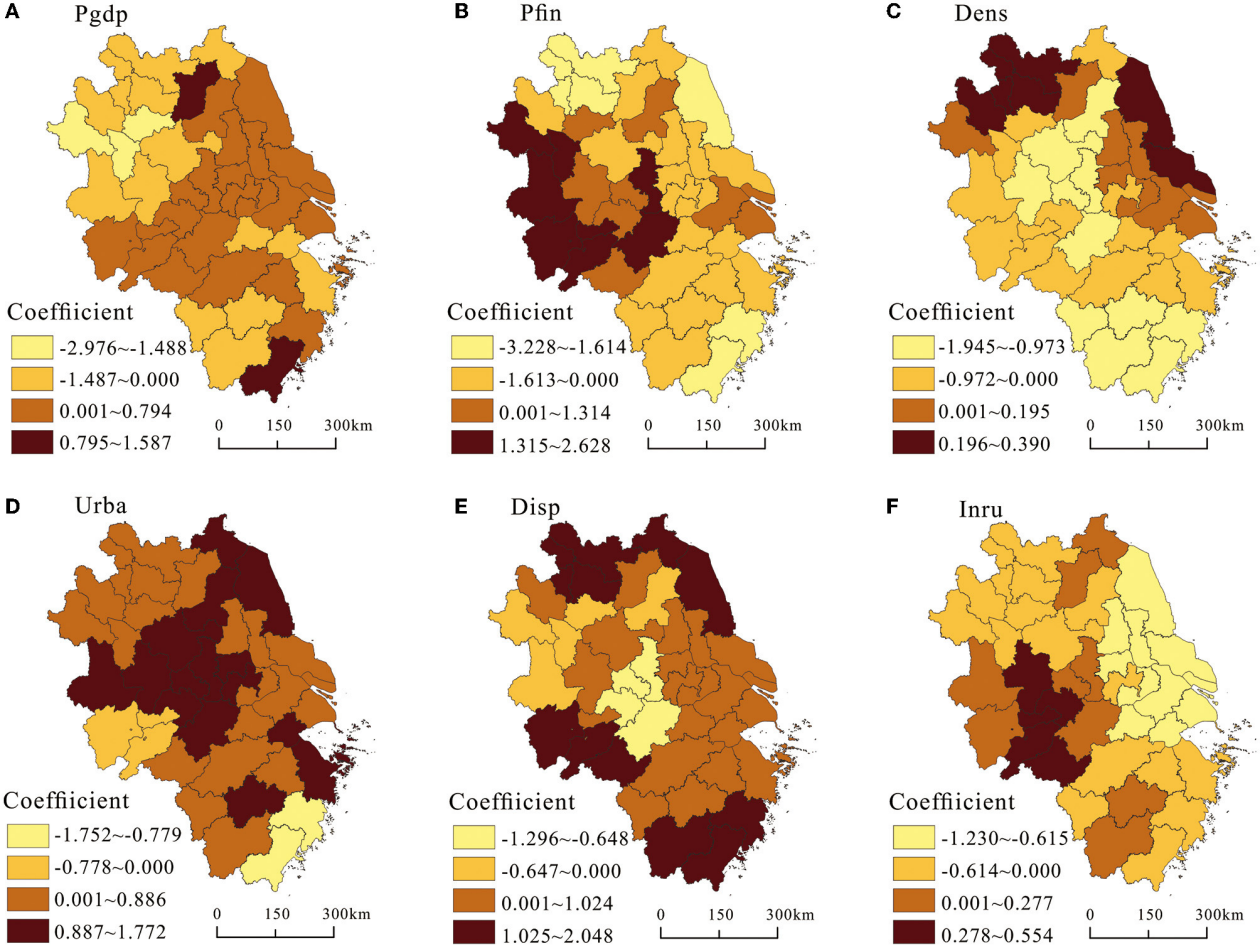
Spatial heterogenous effects of different variables on the supply level of healthcare service.

In order to more institutively explore the differences in the spatial influence of each factor on the supply level of healthcare service, ArcGIS 10.8 software was used to visually express the local regression coefficients. As shown in Figure 7A, Pgdp generally showed a significant positive influence on healthcare service supply with a positive proportion of 58.54% of the total cities and a statistical significance of 51.41%. Although developed economies can afford to pay for expensive healthcare service supply, rapid economic development also created more jobs, attracted more migrants, and increased the demand for healthcare service, which required constant capital injection to ensure and may offset the positive contribution of economic growth to the development of healthcare service. The positive impact of Pgdp on the supply of healthcare service was mainly concentrated in developed cities in southern Jiangsu province and southern Anhui province, and scattered in cities such as Hangzhou, Shaoxing, Taizhou, and Wenzhou, especially in Suqian and Wenzhou which had the largest positive absolute values. The negative impact of Pgdp on healthcare service supply was distributed in northern Anhui province, northern Jiangsu province, and southwestern Zhejiang province, especially Fuyang, Bengbu, and Huainan which had the largest negative absolute values.

As shown in Figure 7B, per capita fiscal expenditure showed a significant negative impact on the supply of healthcare service in general, with a negative proportion of 58.14% of the total number of cities and a statistical significance of 39.40%. The negative impact of Pfin on the supply of healthcare service was mainly distributed in the southern Jiangsu province and northern Zhejiang province. Due to the wider and more comprehensive coverage of productive and non-productive fiscal expenditures in developed cities, the proportion of fiscal expenditures on healthcare service was crowded out to some extent. In addition, due to high population density and high demand for healthcare service in developed cities, the increase in the proportion of fiscal expenditures on healthcare service did not produce a positive marginal improvement effect. In contrast, the positive effect of Pfin on the supply level of healthcare service was mainly concentrated in the western part of the delta, including most cities in Anhui province. In the less developed cities, due to the low fiscal revenue, the fiscal expenditure structure was unreasonable. The fiscal expenditure proportion was unbalanced and more expenditures were tilted to healthcare, education, social security, and employment, which promoted the improvement of the supply level of healthcare service.

As shown in Figure 7C, population density generally exerted a significant negative impact on the supply of healthcare service, with a negative proportion of 65.85% of the total number of cities and a statistical significance of 43.90%. The negative impact of Dens on the supply level of healthcare service was mainly concentrated in the central and southern cities of the delta. This was due to how the increase of Dens stimulated the demand for healthcare resources, and how the relative growth rate of the supply level of healthcare resources was lower than the relative growth rate of the resident population, which put great pressure on the development of urban healthcare service. The positive impact of Dens on the supply level of healthcare service was clustered around the northern Jiangsu province, northern Anhui province, and southern Jiangsu province. This was due to that advanced and sufficient healthcare service resources became important factors for cities to attract population inflow. In addition, with the increasing aging, expectancy, the people's healthcare needs are increasing. These cities strengthened the optimization of the supply-side structure to make full use of healthcare resources to meet social needs and promote the improvement of healthcare service.

As shown in Figure 7D, Urba had a significant positive impact on the supply level of healthcare service, with a positive proportion of 87.80% of the total cities and a statistical significance of 73.17%. The only cities where Urba had a negative impact on the supply level of healthcare service were Taizhou, Wenzhou, Anqing, Chizhou, and Tongling. This was due to the process of urbanization required the support of infrastructure and related public service. The supporting supply of healthcare infrastructure as a public product for people's livelihood would increase accordingly. In addition, with the construction of new-type urbanization is people-centered urbanization. Both the starting and ending points of urbanization were to improve people's quality of life, and a high-quality life required high-quality healthcare service as the foundation and guarantee. Therefore, urbanization had positive impact on the improvement of the supply level of healthcare service in most of cities.

As shown in Figure 7E, per capita disposable income had a significant positive impact on the supply of healthcare service, with a positive proportion of 78.05% of the total number of cities and a statistical significance of 51.97%. The positive effect of Disp on the level of healthcare service supply was mainly distributed in coastal cities and the northern and southern parts of the delta. This was due to that the increase in per capita disposal income led to the increase in consumption demand and consumption capacity, especially with the increase in income level of low-income groups. The share of healthcare expenditure in unit income would continue to increase, thus stimulating and promoting the rapid growth of healthcare resource supply. The negative impact of Disp on the supply level of healthcare service was mainly concentrated in a few cities with low Disp in the central and northwest part of the delta, which weakened demands for healthcare service.

As shown in Figure 7F, urban-rural income gap had a significant negative impact on the supply of healthcare service, with a positive proportion of 65.85% of the total cities and a statistical significance of 54.03%. The negative impact of Inru on the supply level of healthcare service was mainly concentrated in the southern Jiangsu province, the northern Zhejiang province, and the northwestern part of the delta. This was mainly due to the unbalanced allocation of healthcare resources between urban and rural areas, with high-quality healthcare resources concentrated in the central urban areas and insufficient supply of healthcare resources in rural areas. When the income of rural residents increased, they were more inclined to flow to the secondary and tertiary hospitals, resulting in the lagging development of healthcare security and accessibility in rural areas. The positive impact of Inru on the supply level of healthcare service was mainly distributed in the southern part of Anhui province. This was because local governments had increased public healthcare expenditures and supplies to rural areas to avoid widening the income gap between urban and rural areas.

## Discussion

Due to the imbalanced regional economic development, the imbalance of local medical financial capacity, the imbalance of regional income, and the fragmentation of healthcare coverage, the richness of healthcare resource supply in the Yangtze River Delta varies from place to place. The imbalanced supply of healthcare resources has become a serious constraint to the coordinated development and social justice in the Yangtze River Delta region. Although the regional inequality in the supply level of healthcare service in the Yangtze River Delta region has been narrowing and the supply level of healthcare service in each city tends to improve, there are still obvious clusters of spatial differentiation in the supply level of healthcare service, which not only presents cumulative effects in time and inertia effects in practice, but also exhibits stable local spatial autocorrelation structures and specific dependencies or locked paths.

The findings of this study will deepen the understanding of the spatio-temporal heterogeneity of the drivers of healthcare service. In addition, it can provide a practical paradigm for the balanced development of healthcare service levels in other urban agglomerations in China. The supply level of healthcare service is closely related to the current situation of local social and political and economic development, which influences and promotes each other. A favorable social, political, and economic environment that adheres to the principles of efficiency, equity, and optimal planning can promote the optimal allocation of healthcare resources, equalization of healthcare service, and full coverage of healthcare service, while a sound healthcare service system helps to maintain healthy human capital. Especially since the outbreak of the COVID-19, the supply capacity and quality of healthcare service has become an important component of urban quality and an important element in attracting innovative talent. Hence, with the demand of high-quality urban development and people's need for a better life, the improvement of healthcare service level will become the focus of regional competition, which in turn differentiates and reinforces the capacity and quality of regional healthcare service supply.

Admittedly, there are some shortcomings in this study. First, based on broadening the data collection channels, a more comprehensive index system for evaluating the supply level of healthcare service can be established and the research period can be further extended. Second, the heterogenous influence effects of different factors on the supply level of healthcare service could be further diagnosed from different combination of city class, city size, and high-low and equal-unequal types. Third, comparative analysis in terms of provincial, county, and finer dimensions helps to identify the potential spatial sources of the unbalanced supply level of healthcare service.

## Conclusion

Although the equity of the supply of healthcare service has been widely discussed, the regional differences, spatial association patterns, and heterogenous influence effects of the supply of healthcare service has not been systematically studied from the perspective of spatio-temporal association and heterogeneity. Therefore, we analyzed the evolution process and influencing factors of regional healthcare service in 41 cities of the Yangtze River Delta from 2007 to 2019 using spatio-temporal analysis techniques. The main findings are as follows.

(1) In terms of spatio-temporal evolution, the supply level of healthcare service in the Yangtze River Delta region had improved significantly since 2007, and the differences in healthcare service supply between cities had been narrowing without polarization. The supply level of healthcare service in the Yangtze River Delta region generally showed a spatial pattern of “high in the south and low in the north,” with high-level cities highly concentrated in the southern part of Anhui province, southern Jiangsu province, the northern part of Zhejiang province, and low-level cities widely scattered in the northern Anhui province and northern Jiangsu Province. At the same time, it can be found that the higher the supply level of healthcare service, the weaker the inter-annual variation, and the more likely to form the cumulative inertia effect.
(2) In terms of spatio-temporal association, there was an obvious spatial clustering of the supply level of healthcare service in the Yangtze River Delta region, with HH type cities clustered mainly in the southern part of the delta, the LL type cities concentrated in the northern part of the delta, and LH and HL type cities distributed between HH and LL types or at the edge of the delta. The relative lengths of the supply level of healthcare service in the Yangtze River Delta generally showed a heterogenous spatial pattern of low values in the northeast and high values in the southwest. Similarly, relative curvature showed almost the same spatial pattern.
(3) In terms of spatio-temporal influence effects, the temporal impact of Pgdp tended to increase, but did not become positive until 2017, while Pgdp showed a significant positive impact on the supply level of healthcare service across most cities spatially. The temporal impact of Pfin gradually changed from a positive value to a negative value from 2011, and Pfin exhibited a significantly negative impact on the supply level of healthcare service in most cities spatially. The temporal impact of Dens was negative and the intensity of the impact increased, and Dens generally exerted a significantly negative impact on the supply level of healthcare service in most cities in space. The temporal impact of Urba remained positive and the intensity of the impact tended to increase. In addition, Urba showed significantly positive impact on the supply level of healthcare service in the most cities in space. Except for 2007 and 2008, the temporal impact of Disp remained positive, and Disp showed a significant positive impact on the supply level of healthcare service in most cities in space. The temporal impact of Inru kept being negative and maintained a smooth impact intensity, and Inru showed a significant negative impact on the supply level of healthcare service across most cities in space.

To narrow regional development gap of healthcare service, improve the overall efficiency of healthcare spending, and promote high-quality synergistic development of healthcare in the Yangtze River Delta region, relevant policy suggestions have been proposed as follows.

First, local governments should pay more attention to the development of healthcare and improve the performance assessment system of local government healthcare service. On the one hand, local governments should strengthen the performance assessment of the use of government healthcare expenditure funds, focusing on whether the financial investment in healthcare is adequate, timely, and in place, and whether the distribution of healthcare expenditure between urban and rural areas is reasonable. On the other hand, local governments should strengthen the performance appraisal of healthcare institutions and healthcare personnel. In addition, efforts should be made to establish a “five-synergy” basic healthcare system composed of a public health service system, a medical service system, a funding guarantee system, a drug safety supply system, and an industry supervision and law enforcement system so that the general public can enjoy better quality healthcare service.

Second, regional administrative barriers should be broken down and the intra-regional transfer payment system should be improved. The cities in the Yangtze River Delta should strengthen the macro management and regulation of healthcare resources. Within the framework of regional integration, cities with redundant healthcare expenditure scale can appropriately transfer funds to cities with insufficient expenditure scale through the form of transfer payments, which can effectively avoid wasting healthcare resources and make full use of them. Besides, cities with high levels of healthcare service provision should play a supportive role and drive the improvement of basic healthcare service in neighboring cities through resource sharing and other means. Moreover, the internet healthcare mode should be actively explored and promoted to form a healthcare network system and healthcare information data platform covering the entire Yangtze River Delta through the interconnection and sharing of residents' electronic health records and electronic medical record databases.

Third, while pursing rapid economic growth, more economic support should be invested in basic healthcare service to ensure that healthcare service develop in tandem with economic growth by enhancing the economic agglomeration effect and scale effect. Moreover, the positive effect of urbanization on the development of healthcare service could be further amplified by promoting the coordinated urban-rural development and narrowing the income gap between urban and rural residents. In addition, it is necessary to improve the allocation efficiency of healthcare resources per capita by increasing the proportion of financial expenditures on healthcare and upgrading the management and technical level of healthcare expenditure.

## Data Availability Statement

The data could be available upon reasonable requirements through corresponding author.

## Author Contributions

Conceptualization, formal analysis, and writing—review and editing: MH and ZL, methodology: ZL, writing—original draft preparation: MH. Both authors contributed to the article and approved the submitted version.

## Funding

This research was funded by Humanities and Social Sciences Youth Foundation, Ministry of Education of the People's Republic of China, grant number 20YJCZH080, Social Science Foundation of Jiangsu Province, grant number 20SHD009, and the Yangzhou University Qing Lan Project, and the Yangzhou Lv Yang Jinfeng Project in 2020.

## Conflict of Interest

The authors declare that the research was conducted in the absence of any commercial or financial relationships that could be construed as a potential conflict of interest.

## Publisher's Note

All claims expressed in this article are solely those of the authors and do not necessarily represent those of their affiliated organizations, or those of the publisher, the editors and the reviewers. Any product that may be evaluated in this article, or claim that may be made by its manufacturer, is not guaranteed or endorsed by the publisher.
